# Enhancing End-of-Life Care Through Yoga

**DOI:** 10.7759/cureus.54405

**Published:** 2024-02-18

**Authors:** Selvaraj Giridharan, Nandan M Shanbhag

**Affiliations:** 1 Oncology, Tawam Hospital, Al Ain, ARE; 2 College of Medicine and Health Sciences, United Arab Emirates University, Al Ain, ARE; 3 Oncology/Radiation Oncology, Tawam Hospital, Al Ain, ARE; 4 Oncology/Palliative Care, Tawam Hospital, Al Ain, ARE

**Keywords:** symptom management, spiritual well-being, quality of life, holistic approach, end-of-life, yoga, palliative care

## Abstract

This editorial explores the integration of yoga into end-of-life care, emphasizing its potential to enhance the quality of life, comfort, and dignity of patients. Rooted in over 5,000 years of history, yoga’s holistic approach, encompassing physical, mental, and spiritual practices, aligns seamlessly with the goals of end-of-life care. We discuss the benefits of incorporating yoga’s diverse practices, such as physical postures, breathing exercises, and meditation, particularly in palliative care settings. These practices offer significant improvements in physical health, psychological well-being, and spiritual fulfillment, especially pertinent for older adults and patients with serious illnesses like HIV and cancer. The philosophical underpinnings of yoga, emphasizing acceptance, harmony, and peace, provide a framework for a dignified and peaceful transition, resonating deeply with the concept of a ‘good death’.

However, challenges exist in integrating yoga into end-of-life care, including limited research, cultural and religious considerations, physical and emotional limitations of patients, and logistical constraints within healthcare settings. Ethical considerations are also paramount, focusing on patient-centered approaches, respect for individual beliefs, informed consent, and patient autonomy.

The editorial concludes by underscoring the need for further research to evaluate the long-term effects of yoga in end-of-life care and to establish comprehensive ethical guidelines. The integration of yoga offers a multifaceted approach to address not only physical discomfort but also provide emotional and spiritual solace for terminally ill patients, thereby enhancing the overall quality of end-of-life care.

## Editorial

End-of-life care aims to support individuals near the end of their lives comprehensively. It prioritizes comfort, dignity, and quality of life for both patients and their loved ones, and contemporary perspectives emphasize a holistic approach that encompasses medical needs and emotional, spiritual, and social support. It is crucial to understand patients' wishes and involve them in decision-making, managing symptoms, providing pain relief, and addressing psychological and spiritual needs. Palliative care, a critical component of end-of-life care, aims to enhance patients' comfort and quality of life with serious illnesses [1].

Yoga's roots date back over 5,000 years, evolving from ancient texts like the Vedas and later the Yoga Sutras of Patanjali. Initially, it focused on spiritual development, encompassing physical, mental, and spiritual practices. Yoga's philosophy centers on the union of mind, body, and spirit. This offers valuable insights into acceptance, coping mechanisms, and finding peace amidst life's challenges. It emphasizes self-awareness, mindfulness, and achieving balance and harmony. This holistic approach aligns with end-of-life care goals, aiming to address not just physical needs but also emotional and spiritual well-being [2].

This editorial examines the integration of yoga into end-of-life care, highlighting its potential to enhance patients' quality of life, comfort, and dignity.

Benefits

Yoga's holistic practices, including physical postures, breathing exercises, and meditation, offer significant benefits in palliative care, especially for older adults, improving physical health, psychological well-being, and spiritual fulfillment [3]. The adaptability of yoga practices to individual needs in late-life stages underscores its potential to enhance comfort and quality of life at the end of life [4]. Yoga has positively impacted psychological health and quality of life in various patient populations, including those with HIV, highlighting its broad applicability in end-of-life care [5]. Studies show that yoga can particularly benefit cancer patients, improving their physical and emotional well-being, which is crucial in end-of-life scenarios [6]. The philosophical aspects of yoga, emphasizing harmony, transcendence, and death acceptance, resonate with end-of-life care goals, offering a framework for a dignified and peaceful transition.

Challenges

The utilization of yoga in end-of-life care poses several challenges and limitations. Limited research hampers a comprehensive understanding of its specific benefits and limitations in this context. Cultural and religious considerations must be considered, as yoga's association with Indian culture may not be universally appropriate or acceptable. Adequate training, resources, and access to experienced yoga instructors are necessary for healthcare providers to effectively incorporate yoga into end-of-life care. Physical limitations of patients, emotional challenges, time constraints for healthcare providers, patient motivation and adherence, lack of accessibility, integration with conventional care, and ethical considerations impede the implementation of yoga in this setting.

'Good' death

Death is inevitable, but it does not have to be feared or avoided. In fact, according to the ancient wisdom of Yoga, death can be an opportunity for spiritual growth and liberation. Yoga teaches us how to live and die well. Yoga's approach to achieving a 'good' death is deeply rooted in a lifelong spiritual journey. This philosophy, highlighted by yogic leaders, emphasizes that a fulfilling death directly results from a conscious, well-lived life. In yogic practice, one cultivates and directs *Prānā*, or life energy, through the *Sushumnā* in the spine, aiming to release it through the *Brahma*-*Andhra* at the head, symbolizing the ascent of energy through various chakras and the dissolution of earthly ties. Central to this philosophy is *Ashtāngā* Yoga, an eight-fold path encompassing ethical restraints, self-disciplines, physical postures, breath control, and meditation, culminating in *Samadhi*, or union with the universal consciousness. This path is a holistic approach to living, stressing moral conduct, self-control, and deep spiritual practices aimed at liberation from the cycle of rebirth [7].

Ethical considerations

Ethical considerations in implementing yoga in end-of-life care are paramount. Healthcare providers should ensure that yoga interventions are patient-centered, respect individual beliefs, and prioritize informed consent. Healthcare providers should establish ethical guidelines to safeguard patients' autonomy and well-being.

Conclusion

End-of-life care presents significant challenges for healthcare providers. However, integrating yoga as an alternative therapy can offer a holistic approach to care that addresses physical, emotional, and spiritual needs. The potential benefits of yoga in end-of-life care include symptom relief, emotional well-being, and existential support. By incorporating yoga into the care plan, healthcare providers can enhance the quality of life for terminally ill patients and provide them with comfort, peace, and dignity during their final journey.

Researchers should conduct further studies to explore how yoga can be integrated into end-of-life care, assess its long-term effects, and establish ethical guidelines that prioritize patient autonomy and well-being. With its multifaceted approach, yoga addresses physical discomfort and provides emotional and spiritual solace.
